# Network Pharmacology-Based Investigation of the Therapeutic Mechanisms of Action of Danning Tablets in Nonalcoholic Fatty Liver Disease

**DOI:** 10.1155/2021/3495360

**Published:** 2021-04-27

**Authors:** Tong Lin, Li Li, Caijun Liang, Lisheng Peng

**Affiliations:** ^1^The Fourth Clinical Medical School, Guangzhou University of Chinese Medicine, Shenzhen 518033, China; ^2^Shenzhen Hospital of Traditional Chinese Medicine, Shenzhen 518033, China

## Abstract

Nonalcoholic fatty liver disease (NAFLD) is a rising global public health concern due to its prevalence. Danning Tablets (DNt), a composite prescription of Chinese herbal medicine, shows significant curative effects on NAFLD in clinical application. This study aimed to decipher the bioactive substances and potential mechanisms of action of DNt in the treatment of NAFLD, applying an integrated network pharmacology approach. First, the bioactive compounds of DNt were screened based on their pharmacokinetic properties, and the corresponding drug targets were predicted. Then, the NAFLD-related targets were collected. The overlapping targets between the putative targets of DNt and NAFLD-related targets were identified as the potential therapeutic targets of DNt against NAFLD. Subsequently, the networks were constructed and analyzed, and the key bioactive compounds and targets were screened out depending on their importance in the networks. Functional enrichment analysis was carried out to elucidate the potential mechanisms of DNt acting on NAFLD. Finally, a molecular docking simulation was implemented to assess the potential binding affinity between the key targets and the bioactive compounds. As a result, 43 bioactive compounds of DNt and 69 putative targets were identified. Based on the network analysis, we found seven key bioactive compounds (quercetin, *ß*-sitosterol, luteolin, kaempferol, supraene, curcumenolactone C, and stigmasterol) of DNt might treat NAFLD via intervening IL6, MAPK8, VEGFA, CASP3, ALB, APP, MYC, PPARG, and RELA. The functional enrichment analysis revealed that DNt might affect NAFLD by modulating the signaling pathways involved in lipid metabolism, inflammation, oxidation, insulin resistance (IR), atherosclerosis, and apoptosis. Furthermore, most key bioactive compounds might bind firmly with the key targets. This study predicted the multicomponent, multitarget, and multipathway mechanisms of DNt in the treatment of NAFLD from a holistic perspective. DNt could be a promising agent for NAFLD, but further experimental verifications are still needed.

## 1. Introduction

Nonalcoholic fatty liver disease (NAFLD) is presently the most common liver disease, affecting a quarter of the adult population worldwide [1]. NAFLD, hallmarked by lipid accumulation in hepatocytes without significant alcohol intake, encompasses simple hepatic steatosis and nonalcoholic steatohepatitis (NASH), along with cirrhosis and even liver cancer developed by NASH [1]. NASH takes up 25 percent of NAFLD, and more than 1/3 of NASH would die from end-stage liver disease, making it the main reason for liver transplantation [2, 3]. Even though the pathogenesis of NAFLD has not been fully elucidated, insulin resistance (IR), inflammation, and oxidative stress are acknowledged as the essential contributors to the onset and progression of the disease [4, 5]. Currently, insulin sensitizers, antioxidants, lipid-lowering drugs, and lifestyle modifications are the mainly recommended treatment for NAFLD. However, there is no officially approved medication for NAFLD at present, and less than half of the patients can achieve the goal of weight loss [6, 7]. As an important part of complementary and alternative treatments, herbal medicine is a promising candidate for the treatment strategies of chronic liver diseases [8].

Danning Tablet (DNt) is a composite Chinese patent medicine, which consists of seven Chinese herbs: Rhei Radix et Rhizome (Dahuang, RRR), Polygoni Cuspidati Rhizoma et Radix (Huzhang, PCRR), Citri Reticulatae Pericarpium Viride (Qingpi, CRPV), Imperatae Rhizoma (Baimaogen, IR), Citrus Reticulatae Pericarpium (Chenpi, CRP), Curcumae Radix (Yujin, CR), and Crataegi Fructus (Shanzha, CF). DNt has been widely used to treat hepatobiliary diseases, such as cholelithiasis, chronic cholecystitis, and NAFLD for more than ten years in China, and it had obtained a product license by Canadian Natural Medicines and Over-The-Counter Medicines Agency in 2017. A multicenter clinical trial showed that DNt could improve clinical symptoms, serum ALT (alanine aminotransferase) level, and blood lipids of NAFLD patients without significant adverse events [9]. Another two clinical studies reported that DNt could reduce tumor necrosis factor *α* (TNF-*α*) and malondialdehyde, increase superoxide dismutase in serum, and attenuate IR significantly [10, 11]. The experimental studies demonstrated that DNt might alleviate hyperlipidemia, liver function damage, and fatty degeneration and punctiform necrosis of liver tissue in animals with NAFLD [12, 13]. It was also reported that the lipid modulation effects of DNt might be associated with the induction of peroxisomal proliferator-activated receptor *α* (PPAR*α*) and cholesterol 7-hydroxylase (CYP7A1) in rat liver cells [13]. Besides, DNt could exert hepatoprotective effects by regulating oxidative stress, hepatic transporters, hepatic metabolic enzymes, and inflammatory signal pathways [14–16]. However, the essential compounds and the underlying mechanisms of DNt in the treatment of NAFLD have not been elucidated.

Chinese herbal medicine serves as a multicomponent, multitarget, and multipathway therapy, which makes it unique in treating complex diseases, but causes difficulty in mechanism elucidation meanwhile. As an approach integrated systems biology, bioinformatics, and polypharmacology, network pharmacology is a powerful tool to investigate complex diseases and uncover the complex interactions among drugs, targets, and diseases [13–17]. Molecular docking is a widely used *in silico* structure-based method to predict the interactions between molecules and biological targets, which enables the virtual screening of amounts of compounds in a limited time [18].

**Figure 1:**
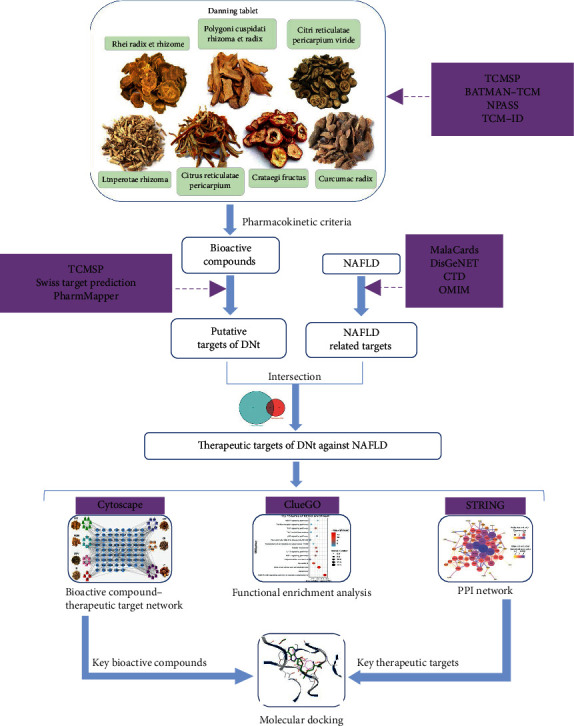
A flowchart of this study.

In this study, a network pharmacology approach was applied to explore the potential mechanisms of DNt against NAFLD. First of all, the bioactive compounds of DNt were screened according to their pharmacokinetic properties, and the corresponding targets of the bioactive compounds were predicted. Then, NAFLD-related targets were collected, the overlapping targets of DNt and NAFLD were regarded as the potential therapeutic targets of DNt against NAFLD. Furthermore, two interactive networks were built to screen out the key bioactive compounds and the key therapeutic targets. After that, functional enrichment analysis was conducted to expound the anti-NAFLD mechanisms of DNt. At last, the binding potentiality between the key target proteins and the bioactive compounds was evaluated through molecular docking. A flowchart of this study is described in Figure 1.

## 2. Materials and Methods

### 2.1. Screening of the Bioactive Compounds of DNt

The information of the compounds of the seven herbs in DNt was collected from Traditional Chinese Medicine Systems Pharmacology Database [19] (TCMSP, http://tcmspw.com/index.php), Bioinformatics Analysis Tool for Molecular Mechanisms of Traditional Chinese Medicine Database (BATMAN-TCM, http://bionet.ncpsb.org/batman-tcm/), Natural Product Activity & Species Source Database (NPASS, http://bidd2.nus.edu.sg/NPASS/), and Traditional Chinese Medicine Information Database [20] (TCM-ID, http://bidd.nus.edu.sg/group/TCMsite/Default.aspx).

Oral bioavailability (OB) is the percentage of an orally administered dose of medicine that reaches the circulatory system; compounds with a higher OB value signify a better capability of being oral drugs [21]. Intestinal epithelial permeability (IEP) measures the intestinal absorption of drugs, which is partly responsible for the bioavailability of oral drugs [22]. Drug-likeness (DL) is a qualitative concept estimating the druggability of compounds, which assesses the compounds' capability of modulating targets [23]. Based on the TCMSP database, OB ≧ 30%, IEP ≧ -0.4, and DL ≧ 0.18 were set as the pharmacokinetic criteria to screen out the bioactive compounds of DNt [24]. The molecule structures of all the bioactive compounds were identified using the PubChem database [25] (https://pubchem.ncbi.nlm.nih.gov/).

### 2.2. Putative Targets of DNt

The putative targets corresponding to the bioactive compounds of DNt were collected from three databases: (1) TCMSP database provides the related targets for the certain compounds; (2) Swiss Target Prediction [26] platform (http://www.swisstargetpredic- tion.ch) predicts targets that have the actives displaying similarity with the query molecule. The species is limited to “*Homo sapiens*,” and only the results with a probability value of >0.7 were collected. (3) PharmMapper [27] platform (http://lilab-ecust.cn/pharmmapper/submitfile.html) predicts targets via a pharmacophore mapping approach; the targets with the setting of “human protein targets only” and a fit score of >3.5 were obtained. After eliminating duplicates, all the predicted targets were converted into Uniprot IDs using UniProtKB database (https://www.uniprot.org/) for the subsequent study.

### 2.3. Therapeutic Targets of DNt against NAFLD

Four disease databases, MalaCards [28] (https://www.malacards.org), DisGeNET [29] (http://www.disgenet.org/), Comparative Toxicogenomics Database [30] (CTD, http://ctdbase.org), and Online Mendelian Inheritance in Man® (OMIM) [31] database (http://www.omim.org/) were used to collect the NAFLD-related targets. The search terms used were “non-alcoholic fatty liver disease” or “NAFLD”.

Based on the preceding steps, two sets of targets had been prepared: the putative targets of DNt and the NAFLD-related targets. Venn diagram package in *R* was used to screen the overlapping targets, which were identified as the potential therapeutic targets of DNt against NAFLD.

### 2.4. Network Construction and Analysis

Two networks were constructed in this study: (1) a bioactive compound-therapeutic target network was built to show the interactions of the bioactive compounds and the therapeutic targets of DNt; (2) a protein-protein interaction (PPI) network was built to explore the interactions of the therapeutic targets of DNt against NAFLD. The PPI analysis was conducted using Search Tool for the Retrieval of Interacting Genes/Proteins platform [32] (STRING, https://string-db.org/), and the criteria were limited in “Homo sapiens” and a high confidence score of 0.7.

The networks were constructed by Cytoscape software version 3.7.2 [33], and the NetworkAnalyzer plug-in was used to analyze the nodes' topological parameters in the network. Two topological parameters, degree and closeness centrality, were calculated to measure the importance of the nodes. A degree value is defined as the number of edges linked to a node. A closeness centrality value is defined as the average of the shortest path length from a node to all other nodes, indicating the closeness of a node to the others in the network [34]. The key compounds and targets were screened out according to their network topological property, which was defined as two times greater than the median degree value and one time greater than the median closeness centrality value of all nodes in the network [35].

### 2.5. Functional Enrichment Analysis

To elucidate the biological mechanisms of therapeutic targets of DNt acting on NAFLD on a systematic level, the ClueGO 2.5.4 plug-in of Cytoscape was used to perform Gene Ontology (GO) and Kyoto Encyclopedia of Genes and Genomes (KEGG) pathway functional enrichment analysis. The minimum gene counts enriched in each GO or KEGG term was set as three, and the terms with *p* values < 0.05 were considered statistically significant. The “ggplot2” package in *R* software (version 4.0.3, the *R* Foundation for Statistical Computing, Vienna, Austria) was used to visualize the GO and KEGG terms.

### 2.6. Molecular Docking Simulation

To evaluate the binding potential of the key bioactive compounds of DNt to key target proteins, molecular docking simulation was performed using GEMDOCK software (version 2.1, Hsinchu, Taiwan) [34]. The empirical scoring function of GEMDOCK is as follows: Fitness = van der Waal energy (vdW) + hydrogen bonding energy (Hbond) + electro statistic energy (Elec). A fitness value was used to estimate the binding affinity of a protein-ligand complex. A lower fitness value indicates a stabler binding between a protein and a ligand. The fitness value of the corresponding protein–original ligand was used as a comparison. And the generic evolutionary method parameters were set as population size = 200, generations = 70, and number of solutions = 2.

All 3D crystal structures of the target protein-ligand complexes were retrieved from Protein Data Bank [35] (PDB, http://www.rcsb.org/pdb/). All 3D molecular structures of the compounds were retrieved from ZINC15 database [36] (http://zinc.docking.org).

## 3. Results

### 3.1. Bioactive Compounds and Putative Targets of DNt

A total of 1303 compounds of DNt were collected from four databases, including 239 compounds in RRR, 188 compounds in PCRR, 54 compounds in CRPV, 64 compounds in IR, 348 compounds in CRP, 315 compounds in CR, and 95 compounds in CF. After excluding duplicates, we finally screened out 43 bioactive compounds with eligible OB, IEP, and DL properties (shown in Supplementary Table 1). What is more, 219 diverse putative targets of these bioactive compounds were obtained from the three target prediction databases.

### 3.2. Bioactive Compound-Therapeutic Target Network

**Figure 2:**
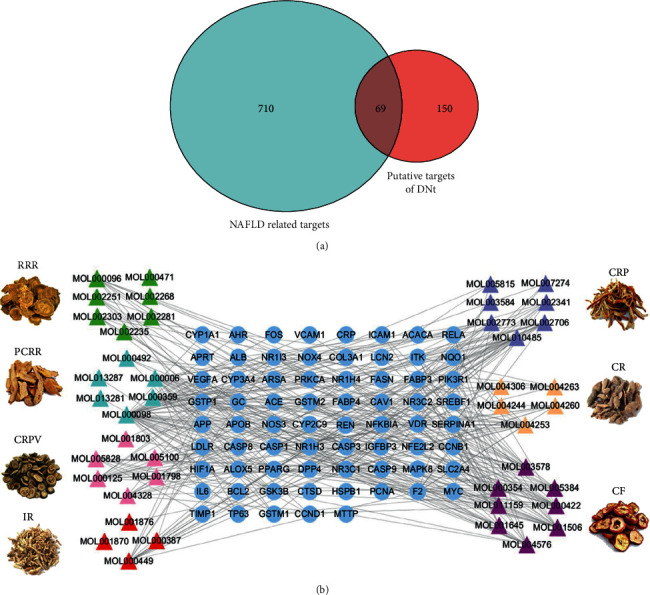
The therapeutic targets of DNt against NAFLD and the bioactive compound-therapeutic target network. (a) The Venn diagram of the overlapping targets between the putative targets of DNt and NAFLD-related targets. (b) The bioactive compound-therapeutic target network of DNt against NAFLD. Triangle nodes represent the bioactive compounds of DNt; circular nodes represent the therapeutic targets; edges represent the relations between compounds and targets. RRR, Rhei Radix et Rhizome; PCRR, Polygoni Cuspidati Rhizoma et Radix; CRPV, Citri Reticulatae Pericarpium Viride; IR, Imperatae Rhizoma; CRP, Citrus Reticulatae Pericarpium; CR, Curcumae Radix; CF, Crataegi Fructus.

We collected a total of 779 NAFLD-related targets from the four databases after the removal of duplicates. And 69 overlapping targets between the putative targets of DNt and NAFLD-related targets were recognized as the therapeutic targets of DNt in the treatment of NAFLD (Figure 2(a) and Supplementary Table 2).

The bioactive compound-therapeutic target network (Figure 2(b)) consisted of 112 nodes (43 bioactive compounds and 69 therapeutic targets) and 356 edges, manifesting a multicompound and multitarget mode of action. Seven bioactive compounds with the highest degree values, quercetin (degree = 41), luteolin (degree = 20), *ß*-sitosterol (degree = 18), curcumenolactone C (degree = 17), supraene (degree = 17), kaempferol (degree = 18), and stigmasterol (degree = 16) were recognized as the key bioactive compounds of DNt against NAFLD, owing to that they were twofold higher than the median degree value of 7 and onefold higher than the median closeness centrality value of 0.37 (Table 1). Higher degree values indicate that these bioactive compounds interact with more therapeutic targets, and higher closeness centrality values indicate their more central position in the network. Additionally, six of the bioactive compounds were from CF, three of them were from PCRR, manifesting the contributions of CF and PCRR in DNt.

### 3.3. Functional Enrichment Analysis

**Figure 3:**
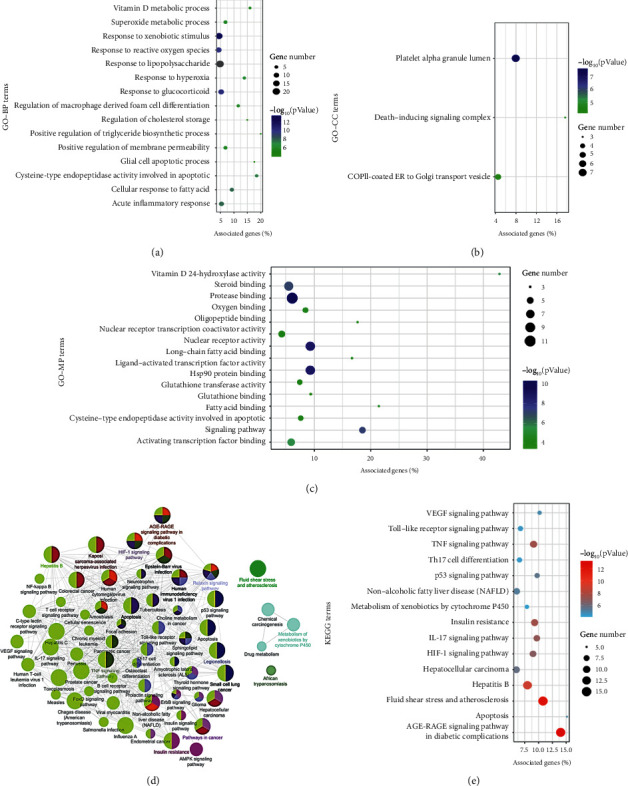
Functional enrichment analysis of the therapeutic targets of DNt against NAFLD. (a) The top 15 GO-BP terms; (b) all the GO-CC terms; (c) the top 15 GO-MF terms. (d) The KEGG pathway enrichment conducted by ClueGO: nodes represent KEGG terms, the node's size represents the enriched significance, and the node's color reflects the enriched classification. (e) The top 15 KEGG pathway terms. Note: the bubble size represents the count of therapeutic targets enriched in a certain pathway; the associated genes (%) mean the percentage of target genes to the background genes of a certain pathway. GO, Gene Ontology; BP, biological process; CC, cellular component; MF, molecule function; KEGG, Kyoto Encyclopedia of Genes and Genomes.

The functional enrichment analysis was performed for the 69 therapeutic targets. In total, 359 GO terms were identified, including 35 molecule function (MF) terms, three cellular component (CC) terms, and 320 biological process (BP) terms. For BP, the therapeutic targets were mainly responsible for the responses to oxidative stress, xenobiotic stimulus, inflammation, vitamin D, and the regulation of lipid, apoptosis, and foam cell differentiation. For MF, the therapeutic targets mainly participated in the binding of protease, fatty acid, steroid, oxygen, glutathione, and the activation of the transcription factor, vitamin D 24-hydroxylase, and cysteine-type endopeptidase involved in apoptosis. For CC, the potential targets were mainly distributed in the endoplasmic reticulum (ER), death-inducing signaling complex, and platelet. The most significantly enriched 15 GO terms are visualized in Figure 3(a)–3(c).

Sixty-one KEGG pathway terms were enriched (Figure 3(d)); among them, signaling pathways of apoptosis, advanced glycation end products (AGE) and receptor for AGE (RAGE), and fluid shear stress and atherosclerosis had the highest proportion of the target genes. The top 15 most significantly enriched terms are shown in Figure 3(e).

### 3.4. PPI Network of the Therapeutic Targets

**Figure 4:**
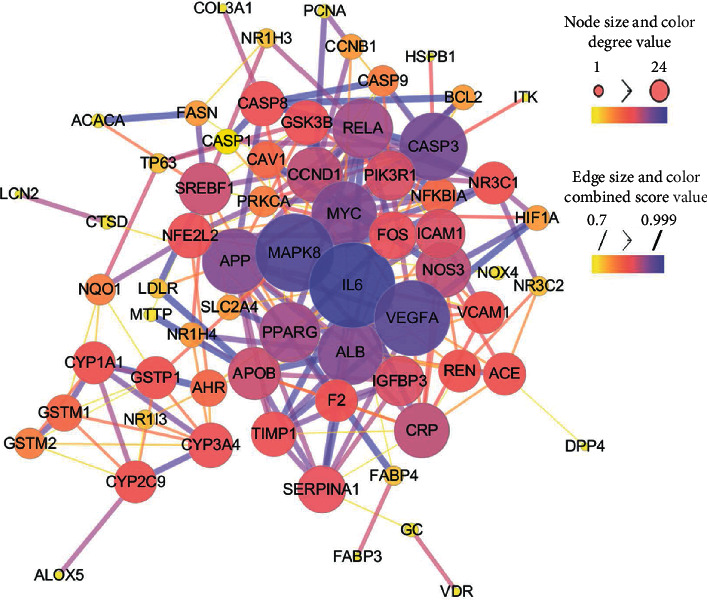
The PPI network of the therapeutic targets of DNt against NAFLD. The size and color of a node are proportional to the degree value, the size and color of an edge are proportional to the combined score. Smaller sizes and brighter colors signify lower degree values.

Because two targets were excluded for the lack of any interaction with other proteins, the PPI network consisted of 235 interactions among 67 therapeutic targets (Figure 4). The median degree value and median closeness centrality value of the PPI network were 6 and 0.31, respectively. Nine proteins, interleukin-6 (IL6), mitogen-activated protein kinase 8 (MAPK8), vascular endothelial growth factor A (VEGFA), caspase-3 (CASP3), Myc proto-oncogene protein (MYC), serum albumin (ALB), amyloid-beta A4 protein (APP), peroxisome proliferator-activated receptor gamma (PPARG), and transcription factor p65 (RELA) met the screening criteria of key nodes in this network, demonstrating their central roles in the pathological processes of NAFLD. These targets acted as irreplaceable mediums to establish the connections between other targets in the development of NAFLD, so they were identified as the key targets of DNt treating NAFLD.  Table 2 lists the detailed information of the key therapeutic targets.

### 3.5. Molecular Docking Simulation

**Figure 5:**
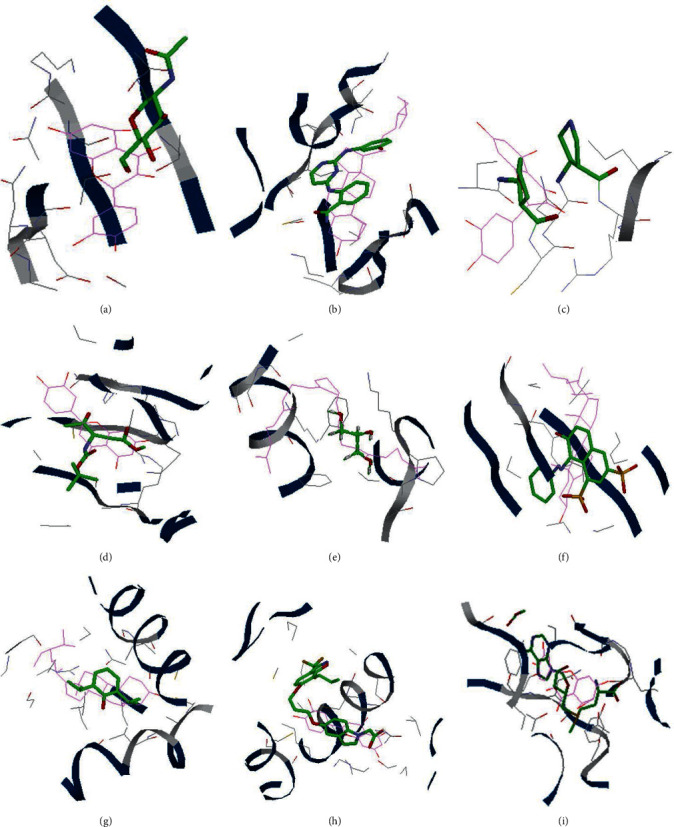
Molecular docking models of the key target proteins with the key bioactive compounds. (a) IL6-quercetin, (b) MAPK8-stigmasterol, (c) VEGFA-quercetin, (d) CSP3-luteolin, (e) MYC- supraene, (f) APP-stigmasterol, (g) ALB-stigmasterol, (h) PPARG-luteolin, and (i) RELA-quercetin. The original ligands are labeled in green color, and the predicted poses of the bioactive compounds are labeled in pink color.

Nine key target proteins, IL6, MAPK8, VEGFA, CASP3, MYC, APP, ALB, PPARG, and RELA, were docked with seven key bioactive compounds, quercetin, luteolin, *ß*-sitosterol, curcumenolactone C, supraene, kaempferol, and stigmasterol. The results found that 30 target protein–bioactive compound complexes (47.62% of all) might bind firmer than the corresponding protein–original ligand complexes, indicating that numerous key bioactive compounds might bind closely with the key therapeutic targets to exercise regulatory effects. Among all, the RELA-quercetin complex had the best fitness value of −113.07 Kcal/mol, followed by ALB-stigmasterol (−111.65 Kcal/mol) and RELA-luteolin (−110.22 Kcal/mol).  Table 3 shows the results of docking, and the docking model of every certain key target and the corresponding best-binding bioactive compound is shown in Figure 5.

## 4. Discussion

In the present study, the multicompound, multitarget, multipathway mechanisms of action of DNt against NAFLD were clarified applying the network pharmacology approach.

First of all, the bioactive compounds and the putative targets of DNt were identified, and the overlapping targets between DNt and NAFLD were recognized as the therapeutic targets of DNt against NAFLD (Figure 2(a)). Quercetin, *ß*-sitosterol, luteolin, kaempferol, supraene, curcumenolactone C, and stigmasterol were selected as the key bioactive compounds of DNt treating NAFLD, based on their contributions in the bioactive compound-therapeutic target network (Figure 2(b) and Table 1). Quercetin, luteolin, and kaempferol are all members of the flavone family, which are widely found in vegetables, fruits, and herbs. The flavones display extensive pharmacological activities, including anti-inflammatory, antioxidative, antimicrobial, antiapoptotic, hepatoprotective, and anticancer [37]. Quercetin could ameliorate NAFLD progression by mitigating oxidative stress, inflammation, and lipid metabolic disorders [38]. It could also modulate the intestinal probiotics and sequentially regulate gut-liver axis activation in NAFLD [39]. Luteolin could relieve diet-induced obesity and its metabolic complications, such as adiposopathy, hepatic steatosis, and IR, by regulating PPARG [40] and Toll-like receptor signaling pathways [41]. Luteolin and kaempferol could both abrogate lipid accumulation induced by the activation of liver *X* receptor- (LXR-) sterol regulatory element-binding protein 1c (SREBP-1c) pathway to mitigate hyperlipemia, NAFLD, and other components of metabolic syndrome (MetS) [42–44].

Supraene, also known as squalene, is a triterpenoid performing anticancer, antioxidant, and detoxicant bioactivities. Supraene had anticholesterolemia and antiatherosclerotic effects through the transactivation of LXR but without the alteration of SREBP-1c expression [45]. *ß*-sitosterol and stigmasterol are two kinds of the most occurring phytosterols, which can decrease the absorption of dietary lipids and bile acids and attenuate hepatic lipid accumulation and weight gain [46, 47]. Curcumenolactone is a carabrane-type sesquiterpene lactones. A previous study showed curcumenolactones A, B, C could protect the liver from D-galactosamine-induced acute injury in mice [48]. To conclude, three flavonoids (quercetin, luteolin, and kaempferol), three natural lipids (*β*-sitosterol, supraene, stigmasterol), and one sesquiterpene lactone undertake the pharmacology basis of the anti-NAFLD effects of DNt, which were consistent with the pathogenesis of promotion of NAFLD.

When it comes to herbs, CF and PCRR stood out for their major contributions of providing more bioactive compounds. In a systematic review, CF was the most frequently used herb in the treatment of NAFLD [49], the extract of which could significantly enhance the activities of antioxidant enzymes, lipid metabolism enzymes, and fatty acid oxidation-related enzymes in the liver of high-fat diet-fed mice [50]. The PCRR extract could inhibit cell proliferation, cell migration, and vessel formation in endothelial cells by suppressing VEGF-induced activations of c-Jun N-terminal kinase (JNK) and reactive oxygen species (ROS) [51]. As for the other component herbs, the citrus flavonoids isolated from CRP might exert hepatoprotective and anti-inflammatory effects by suppressing nuclear factor-*κ*B (NF-*κ*B) and MAPK signaling pathways in rats with NAFLD [52]. RRR was reported to show liver-protective function [53]; the extract of IR showed antioxidation ability *in vitro* [54]; the combined treatment with CR and Glycyrrhizae Radix et Rhizoma could inhibit body weight gain, lipid metabolic disturbances, serum liver enzymes, and hepatic steatosis in rats [55].

Then, the functional enrichment analysis of the therapeutic targets demonstrated the mechanisms of DNt against NAFLD might be focused on apoptosis-associated pathways, MetS-associated pathways (e.g., AGE-RAGE, IR, and atherosclerosis pathways), inflammation-associated pathways (e.g., IL-17, TNF, hypoxia-inducible factor-1 (HIF-1), and Toll-like pathways), and cancer-associated pathways (e.g., VEGF and p53 pathways) (Figure 3). As components of MetS, NAFLD and atherosclerosis share various pathophysiological mechanisms, including inflammation, oxidative stress, unbalanced coagulation-fibrinolysis, chronic intermittent hypoxia, altered adipokine profile, and unfavorable lipidosis [56]. AGEs accelerate the production of RAGEs and the subsequent AGE-RAGE interactions contribute to hepatic fat accumulation, giving rise to inflammation, IR, fibrosis, and other complications of NAFLD [57].

After that, IL6, MAPK8, VEGFA, CASP3, MYC, APP, ALB, PPARG, and RELA were identified as the key targets of DNt against NAFLD, due to their high degree and closeness centrality values (Figure 4). These targets interacted broadly and closely with other targets, indicating their central roles in the disease network. IL-6 has been acknowledged as a central factor in liver inflammation. The activation of IL-6 signaling pathway can induce the production of acute-phase proteins, such as fibrinogen and haptoglobin, leading to hepatitis syndrome, cholestasis subtype, fibrosis, and worse [58]. The activation of MAPK8, also named JNK1, participates in fat-induced apoptosis and IR, and the latter would propel NAFLD to more advanced stages [59]. VEGFA is a sentinel regulator of angiogenesis. It is widely believed that angiogenesis is correlated with NAFLD and fibrogenic progression [60]. For example, serum VEGFA and VEGFR1 levels were significantly elevated in NAFLD patients, and the borderline was even higher in NASH patients [61].

Among the key targets, CASP3 is well-known for its irreplaceable role in the apoptosis process, which can promote inflammation, IR, fibrosis, and cirrhosis in turn [62]. RELA is one protein of the NF-*κ*B family. Saturated fatty acids can activate NF-*κ*B and MYC signaling pathways, followed by increased proinflammatory cytokines, such as TNF-*α* and IL-6, resulting in the development of IR and NAFLD [63, 64]. APP is the precursor protein of amyloid-*β*, which is a consequence of IR and is widely known as a key pathological hallmark of Alzheimer's disease [65]. Moreover, APP can also promote IR in the liver in turn [66]. ALB acts as a key carrier in plasma, possessing the ability of binding with free fatty acids, inflammatory mediators, ROS, lipopolysaccharide, and bacterial antigens, which enable it to perform a vast series of regulatory activity in inflammation, antioxidation, and endothelial function [67, 68]. PPARG is one isotype of PPARs, which is pivotal for the lipogenesis in hepatocytes. Therefore, PPARG is regarded as a characteristic of NAFLD [69]. Contradictorily, PPARG also possesses anti-inflammatory properties, especially in macrophages [70], which might limit hepatic stellate cell proliferation and subsequent fibrosis [71]. Thus, it is undoubted that PPARG plays a critical role in NAFLD, but its multiple actions are still controversial. To sum up, it was speculated that DNt might perform integrative functions of lipid mediation, anti-inflammation, antioxidant, anti-IR, antiapoptosis, and antiendothelial/fibrosis on NAFLD through key targets in associated signaling pathways.

In the end, the molecular docking (Table 3 and Figure 5) showed that about half of the key bioactive compounds might bind firmly to the key target proteins, which provided the possibility of compound-target combination and pharmacological activities realization. The molecular docking simulation should strengthen the ratiocinations in this study, to some extent, but experimental validations are still needed.

## 5. Conclusions

This study applied a network pharmacology approach to elucidate the underlying mechanisms of DNt against NAFLD in a holistic manner. Seven bioactive compounds of DNt, quercetin, *ß*-sitosterol, luteolin, kaempferol, supraene, curcumenolactone C, and stigmasterol, might exert the crucial pharmacological effects. Moreover, IL6, MAPK8, VEGFA, CASP3, MYC, APP, ALB, PPARG, and RELA might be the key targets regulated by those bioactive compounds. Besides, signaling pathways of apoptosis, inflammation, oxidant stress, IR, and lipid metabolism should be mainly involved. This study may inspire insights into novel therapeutic strategies of NAFLD and provide references for future researches. Nevertheless, further experiments are still required to validate the findings of this study.

## Figures and Tables

**Table 1:** The information of the key bioactive compounds of DNt.

TCMSP ID	Name	Molecular formula	Structural formula	OB (%)	IEP	DL	Source	Degree value	Closeness centrality value
MOL000098	Quercetin	C_15_H_10_O_7_	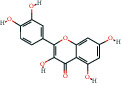	46.43	0.05	0.28	PCRR, CF	41	0.53
MOL000006	Luteolin	C_15_H_10_O_6_	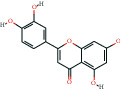	36.16	0.19	0.25	PCRR, CF	20	0.43
MOL000359	*β*-sitosterol	C_29_H_50_O	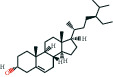	36.91	1.32	0.75	PCRR, IR, CRP, RRR, CR, CF	18	0.43
MOL004253	Curcumenolactone C	C_15_H_20_O_4_	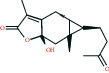	39.7	0.16	0.19	CR	17	0.43
MOL001506	Supraene	C_30_H_50_	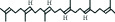	33.55	2.08	0.42	CF	17	0.41
MOL000422	Kaempferol	C_15_H_10_O_6_	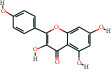	41.88	0.26	0.24	CF	17	0.40
MOL000449	Stigmasterol	C_29_H_48_O	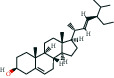	43.83	1.44	0.76	IR, CF	16	0.42

OB, oral bioavailability; IEP, intestinal epithelial permeability; DL, drug-likeness.

**Table 2:** The key therapeutic targets of DNt in the treatment of NAFLD.

No.	Target name	Gene symbol	Uniprot ID	Degree value	Closeness centrality value
1	Interleukin-6	IL6	P05231	24	0.55
2	Mitogen-activated protein kinase 8	MAPK8	P45983	21	0.53
3	Vascular endothelial growth factor A	VEGFA	P15692	20	0.52
4	Caspase-3	CASP3	P42574	17	0.50
5	Myc proto-oncogene protein	MYC	P01106	16	0.49
6	Amyloid-beta A4 protein	APP	P05067	16	0.50
7	Serum albumin	ALB	P02768	16	0.48
8	Peroxisome proliferator-activated receptor gamma	PPARG	P37231	14	0.47
9	Transcription factor p65	RELA	Q04206	14	0.47

**Table 3:** The results of molecular docking simulation.

Protein (PDB ID)	Ligand	Fitness (Kcal/mol)	Protein (PDB ID)	Ligand	Fitness (Kcal/mol)
IL6 (5FUC)	NAG^*∗*^	−82.47	MAPK8 (2GMX)	877^*∗*^	−105.32
	Quercetin	−96.46		Stigmasterol	−103.52
	Kaempferol	−94.28		*β*-Sitosterol	−102.45
	Supraene	−93.65		Supraene	−98.66
	Luteolin	−86.82		Luteolin	−90.16
	Stigmasterol	−83.16		Curcumenolactone C	−88.64
	Curcumenolactone C	−78.64		Quercetin	−80.01
	*β*-Sitosterol	−78.38		Kaempferol	−78.28

VEGFA (6D3O)	XCP^*∗*^	−74.42	CSP3 (3GJR)	DZE^*∗*^	−64.24
	Quercetin	−81.67		Luteolin	−109.76
	Kaempferol	−81.48		Quercetin	−101.75
	Curcumenolactone C	−65.03		*β*-Sitosterol	−93.91
	Supraene	−62.96		Kaempferol	−92.97
	Luteolin	−62.85		Stigmasterol	−90.54
	Stigmasterol	−53.81		Supraene	−85.62
	*β*-Sitosterol	−51.30		Curcumenolactone C	−82.71

MYC (5I4Z)	GOL^*∗*^	v42.74	APP (3OVJ)	ORA^*∗*^	−89.52
	Supraene	−102.18		Stigmasterol	−82.77
	*β*-Sitosterol	−89.16		*β*-Sitosterol	−80.19
	Stigmasterol	−85.25		Supraene	−76.58
	Luteolin	−83.55		Quercetin	−69.45
	Quercetin	−78.06		Luteolin	−68.01
	Kaempferol	−73.66		Kaempferol	−66.05
	Curcumenolactone C	−71.46		Curcumenolactone C	−59.21

ALB (1E7A)	PFL^*∗*^	−69.57	PPARG (2ATH)	3EA^*∗*^	−97.19
	Stigmasterol	−111.65		Luteolin	−99.33
	Luteolin	−107.49		Stigmasterol	−97.79
	*β*-Sitosterol	−100.86		*β*-Sitosterol	−95.81
	Curcumenolactone C	−93.57		Supraene	−93.44
	Supraene	−90.49		Kaempferol	−93.44
	Quercetin	−88.94		Quercetin	−91.09
	Kaempferol	−86.36		Curcumenolactone C	−83.09

RELA (3QXY)	SAM^*∗*^	−142.70	RELA (3QXY)	Kaempferol	−107.02
	Quercetin	−113.07		*β*-Sitosterol	−95.78
	Luteolin	−110.22		Stigmasterol	−94.77
	Supraene	−107.85		Curcumenolactone C	−83.78

^*∗*^NAG, 877, XCP, DZE, GOL, ORA, PFL, 3EA, and SAM are the names of original ligands for corresponding targets in the PDB database.

## Data Availability

All the data used to support the findings of this study are included in the article or could be accessed in the open online databases mentioned in the Methods section.

## Supplementary Materials

Supplementary Materials Supplementary Table 1: information of all the bioactive compounds of DNt. Supplementary Table 2: the potential targets of DNt in the treatment of NAFLD
