# Supplementary material for: Network Pharmacology-Based Investigation of the Therapeutic Mechanisms of Action of Danning Tablets in Nonalcoholic Fatty Liver Disease
Source: Evid Based Complement Alternat Med. 2021 Apr 27;2021:3495360. doi: 10.1155/2021/3495360 (PMC8096548; doi:10.1155/2021/3495360)
Supplement: Supplementary Materials — Supplementary Table 1: information of all the bioactive compounds of DNt. Supplementary Table 2: the potential targets of DNt in the treatment of NAFLD [file 3495360.f1.zip › 3495360.f1/Supplementary Table 1.docx]

**Supplementary Table 1**: Information of all the bioactive compounds of DNt.

| No. | TCMSP ID | Name | OB  (%) | IEP | DL | Source | Degree | Closeness centrality |
| --- | --- | --- | --- | --- | --- | --- | --- | --- |
| 1 | MOL002235 | Eupatin | 50.8 | 0.53 | 0.41 | RRR | 26 | 0.38 |
| 2 | MOL002251 | Mutatochrome | 48.64 | 1.97 | 0.61 | RRR | 17 | 0.36 |
| 3 | MOL002268 | Rhein | 47.07 | -0.2 | 0.28 | RRR, PCRR | 5 | 0.35 |
| 4 | MOL002281 | Toralactone | 46.46 | 0.86 | 0.24 | RRR | 5 | 0.32 |
| 5 | MOL002303 | Palmidin A | 32.45 | -0.36 | 0.65 | RRR | 12 | 0.35 |
| 6 | MOL000471 | Aloe-emodin | 83.38 | -0.12 | 0.24 | RRR | 3 | 0.34 |
| 7 | MOL000096 | (-)-Catechin | 49.68 | -0.03 | 0.24 | RRR | 28 | 0.38 |
| 8 | MOL013287 | Physovenine | 106.21 | 0.51 | 0.19 | PCRR | 17 | 0.36 |
| 9 | MOL013281 | 6,8-Dihydroxy-7-Methoxyxanthone | 35.83 | 0.68 | 0.21 | PCRR | 8 | 0.35 |
| 10 | MOL000492 | (+)-Catechin | 54.83 | -0.03 | 0.24 | PCRR | 18 | 0.37 |
| 11 | MOL000006 | Luteolin | 36.16 | 0.19 | 0.25 | PCRR, CF | 46 | 0.40 |
| 12 | MOL000098 | Quercetin | 46.43 | 0.05 | 0.28 | PCRR, CF | 101 | 0.48 |
| 13 | MOL000359 | β-sitosterol | 36.91 | 1.32 | 0.75 | PCRR, IR, CRP, RRR, CR, CF | 52 | 0.41 |
| 14 | MOL004328 | Naringenin | 59.29 | 0.28 | 0.21 | CRPV, CRP, CR | 22 | 0.37 |
| 15 | MOL001798 | Neohesperidin_qt | 71.17 | 0.26 | 0.27 | CRPV | 9 | 0.35 |
| 16 | MOL005100 | 5,7-Dihydroxy-2-(3-hydroxy-4-methoxyphenyl)chroman-4-one | 47.74 | 0.28 | 0.27 | CRPV, CRP | 18 | 0.37 |
| 17 | MOL001803 | Sinensetin | 50.56 | 1.12 | 0.45 | CRPV, CRP | 35 | 0.39 |
| 18 | MOL005828 | Nobiletin | 61.67 | 1.05 | 0.52 | CRPV, CRP | 29 | 0.38 |
| 19 | MOL000125 | (-)-Alpha-pinene | 46.25 | 1.85 | 0.05 | CRPV | 35 | 0.39 |
| 20 | MOL001870 | Luteolinidin | 53.66 | 0.4 | 0.22 | IR | 2 | 0.33 |
| 21 | MOL000387 | Bifendate | 31.1 | 0.15 | 0.67 | IR | 32 | 0.38 |
| 22 | MOL000449 | Stigmasterol | 43.83 | 1.44 | 0.76 | IR, CF | 60 | 0.42 |
| 23 | MOL001876 | 6-Methoxyflavone | 34.56 | 1.11 | 0.18 | IR | 12 | 0.35 |
| 24 | MOL005815 | Citromitin | 86.9 | 0.88 | 0.51 | CRP | 22 | 0.37 |
| 25 | MOL010485 | Icosapent | 45.66 | 1.34 | 0.21 | CRP | 40 | 0.39 |
| 26 | MOL007274 | Skrofulein | 30.35 | 0.72 | 0.3 | CRP | 24 | 0.37 |
| 27 | MOL003584 | Xanthoxyletin | 35.51 | 0.89 | 0.21 | CRP | 15 | 0.36 |
| 28 | MOL002773 | β-Carotene | 37.18 | 2.25 | 0.58 | CRP | 11 | 0.33 |
| 29 | MOL002341 | Hesperetin | 70.31 | 0.37 | 0.27 | CRP | 33 | 0.38 |
| 30 | MOL002706 | Phytoene | 39.56 | 2.22 | 0.5 | CRP | 38 | 0.39 |
| 31 | MOL004244 | Curcolonol | 59.52 | 0.15 | 0.2 | CR | 2 | 0.33 |
| 32 | MOL004253 | Curcumenolactone C | 39.7 | 0.16 | 0.19 | CR | 60 | 0.42 |
| 33 | MOL004260 | (E)-1,7-Diphenyl-3-hydroxy-1-hepten-5-one | 64.66 | 0.93 | 0.18 | CR | 24 | 0.37 |
| 34 | MOL004263 | (E)-5-Hydroxy-7-(4-hydroxyphenyl)-1-phenyl-1-heptene | 46.9 | 1.04 | 0.19 | CR | 24 | 0.36 |
| 35 | MOL004306 | Zedoalactone B | 103.59 | -0.08 | 0.22 | CR | 6 | 0.35 |
| 36 | MOL005384 | Suchilactone | 57.52 | 0.82 | 0.56 | CF | 40 | 0.39 |
| 37 | MOL001645 | Linoleyl Acetate | 42.1 | 1.36 | 0.2 | CF | 30 | 0.38 |
| 38 | MOL004576 | (+)-Taxifolin | 57.84 | -0.23 | 0.27 | CF | 51 | 0.41 |
| 39 | MOL003578 | Cycloartenol | 38.69 | 1.53 | 0.78 | CF | 39 | 0.38 |
| 40 | MOL011159 | Ergosta-4,6,8(14),22-tetraene-3-one | 48.32 | 1.51 | 0.75 | CF | 34 | 0.38 |
| 41 | MOL001506 | Supraene | 33.55 | 2.08 | 0.42 | CF | 44 | 0.40 |
| 42 | MOL000354 | Isorhamnetin | 49.6 | 0.31 | 0.31 | CF | 36 | 0.39 |
| 43 | MOL000422 | Kaempferol | 41.88 | 0.26 | 0.24 | CF | 42 | 0.40 |

Notes: OB, oral bioavailability; IEP, intestinal epithelial permeability; DL, drug-likeness.
