# Supplementary material for: Network Pharmacology-Based Investigation of the Therapeutic Mechanisms of Action of Danning Tablets in Nonalcoholic Fatty Liver Disease
Source: Evid Based Complement Alternat Med. 2021 Apr 27;2021:3495360. doi: 10.1155/2021/3495360 (PMC8096548; doi:10.1155/2021/3495360)
Supplement: Supplementary Materials — Supplementary Table 1: information of all the bioactive compounds of DNt. Supplementary Table 2: the potential targets of DNt in the treatment of NAFLD [file 3495360.f1.zip › 3495360.f1/Supplementary Table 2.docx]

**Supplementary Table 2**: The potential targets of DNt in the treatment of NAFLD.

| No. | Target name | Gene symbol | Uniprot ID | PPI network degree | PPI network CC |
| --- | --- | --- | --- | --- | --- |
| 1 | Interleukin-6 | IL6 | P05231 | 24 | 0.55 |
| 2 | Mitogen-activated protein kinase 8 | MAPK8 | P45983 | 21 | 0.53 |
| 3 | Vascular endothelial growth factor A | VEGFA | P15692 | 20 | 0.52 |
| 4 | Caspase-3 | CASP3 | P42574 | 17 | 0.50 |
| 5 | Myc proto-oncogene protein | MYC | P01106 | 16 | 0.49 |
| 6 | Amyloid beta A4 protein | APP | P05067 | 16 | 0.50 |
| 7 | Serum albumin | ALB | P02768 | 16 | 0.48 |
| 8 | Peroxisome proliferator-activated receptor gamma | PPARG | P37231 | 14 | 0.47 |
| 9 | Transcription factor p65 | RELA | Q04206 | 14 | 0.47 |
| 10 | C-reactive protein | CRP | P02741 | 12 | 0.42 |
| 11 | G1/S-specific cyclin-D1 | CCND1 | P24385 | 12 | 0.46 |
| 12 | Nitric oxide synthase, endothelial | NOS3 | P29474 | 12 | 0.44 |
| 13 | Sterol regulatory element-binding protein 1 | SREBF1 | P36956 | 11 | 0.43 |
| 14 | Apolipoprotein B-100 | APOB | P04114 | 11 | 0.43 |
| 15 | Phosphatidylinositol 3-kinase regulatory subunit alpha | PIK3R1 | P27986 | 10 | 0.43 |
| 16 | Intercellular adhesion molecule 1 | ICAM1 | P05362 | 10 | 0.44 |
| 17 | Insulin-like growth factor-binding protein 3 | IGFBP3 | P17936 | 10 | 0.45 |
| 18 | Alpha-1-antitrypsin | SERPINA1 | P01009 | 9 | 0.41 |
| 19 | Proto-oncogene c-Fos | FOS | P01100 | 9 | 0.44 |
| 20 | Nuclear factor erythroid 2-related factor 2 | NFE2L2 | Q16236 | 8 | 0.43 |
| 21 | Cytochrome P450 3A4 | CYP3A4 | P08684 | 8 | 0.35 |
| 22 | Metalloproteinase inhibitor 1 | TIMP1 | P01033 | 8 | 0.41 |
| 23 | Glycogen synthase kinase-3 beta | GSK3B | P49841 | 8 | 0.42 |
| 24 | Caspase-8 | CASP8 | O15519 | 8 | 0.42 |
| 25 | Glutathione S-transferase P | GSTP1 | P09211 | 7 | 0.39 |
| 26 | Vascular cell adhesion protein 1 | VCAM1 | P19320 | 7 | 0.42 |
| 27 | Renin | REN | P00797 | 7 | 0.40 |
| 28 | Angiotensin-converting enzyme | ACE | P12821 | 7 | 0.38 |
| 29 | Prothrombin | F2 | P00734 | 7 | 0.40 |
| 30 | Cytochrome P450 2C9 | CYP2C9 | P11712 | 7 | 0.30 |
| 31 | Glucocorticoid receptor | NR3C1 | P04150 | 7 | 0.40 |
| 32 | Cytochrome P450 1A1 | CYP1A1 | P04798 | 7 | 0.34 |
| 33 | Glutathione S-transferase Mu 1 | GSTM1 | P09488 | 6 | 0.31 |
| 34 | Caveolin-1 | CAV1 | Q03135 | 6 | 0.40 |
| 35 | Aryl hydrocarbon receptor | AHR | P35869 | 6 | 0.42 |
| 36 | NF-kappa-B inhibitor alpha | NFKBIA | P25963 | 6 | 0.40 |
| 37 | NAD(P)H dehydrogenase [quinone] 1 | NQO1 | P15559 | 5 | 0.33 |
| 38 | Protein kinase C alpha type | PRKCA | P17252 | 5 | 0.38 |
| 39 | Glutathione S-transferase Mu 2 | GSTM2 | P28161 | 5 | 0.30 |
| 40 | Caspase-9 | CASP9 | P55211 | 5 | 0.40 |
| 41 | G2/mitotic-specific cyclin-B1 | CCNB1 | P14635 | 4 | 0.36 |
| 42 | Bile acid receptor | NR1H4 | Q96RI1 | 4 | 0.39 |
| 43 | Solute carrier family 2, facilitated glucose transporter member 4 | SLC2A4 | P14672 | 4 | 0.42 |
| 44 | Caspase-1 | CASP1 | P29466 | 4 | 0.40 |
| 45 | Fatty acid synthase | FASN | P49327 | 4 | 0.33 |
| 46 | Apoptosis regulator Bcl-2 | BCL2 | P10415 | 4 | 0.39 |
| 47 | Hypoxia-inducible factor 1-alpha | HIF1A | Q16665 | 4 | 0.39 |
| 48 | Nuclear receptor subfamily 1 group I member 3 | NR1I3 | Q14994 | 3 | 0.31 |
| 49 | Tumor protein 63 | TP63 | Q9H3D4 | 3 | 0.35 |
| 50 | Oxysterols receptor LXR-alpha | NR1H3 | Q13133 | 3 | 0.35 |
| 51 | Mineralocorticoid receptor | NR3C2 | P08235 | 3 | 0.33 |
| 52 | Fatty acid-binding protein, adipocyte | FABP4 | P15090 | 3 | 0.37 |
| 53 | Low-density lipoprotein receptor | LDLR | P01130 | 3 | 0.36 |
| 54 | NADPH oxidase 4 | NOX4 | Q9NPH5 | 2 | 0.36 |
| 55 | Vitamin D-binding protein | GC | P02774 | 2 | 0.30 |
| 56 | Cathepsin D | CTSD | P07339 | 2 | 0.34 |
| 57 | Proliferating cell nuclear antigen | PCNA | Q15004 | 2 | 0.32 |
| 58 | Acetyl-CoA carboxylase 1 | ACACA | Q13085 | 2 | 0.31 |
| 59 | Microsomal triglyceride transfer protein large subunit | MTTP | P55157 | 2 | 0.34 |
| 60 | Dipeptidyl peptidase 4 | DPP4 | P27487 | 1 | 0.27 |
| 61 | Tyrosine-protein kinase ITK/TSK | ITK | Q08881 | 1 | 0.30 |
| 62 | Heat shock protein beta-1 | HSPB1 | P04792 | 1 | 0.34 |
| 63 | Fatty acid-binding protein, heart | FABP3 | P05413 | 1 | 0.27 |
| 64 | Vitamin D3 receptor | VDR | P11473 | 1 | 0.23 |
| 65 | Collagen alpha-1(III) chain | COL3A1 | P02461 | 1 | 0.30 |
| 66 | Neutrophil gelatinase-associated lipocalin | LCN2 | P80188 | 1 | 0.25 |
| 67 | Arachidonate 5-lipoxygenase | ALOX5 | P09917 | 1 | 0.23 |
| 68 | Arylsulfatase A | ARSA | P15289 | - | - |
| 69 | Adenine phosphoribosyltransferase | APRT | P07741 | - | - |

Note: PPI, protein-protein interaction.
